# Transformation of the Hydrate of Chloral into Chloroform in the Human Organism

**Published:** 1870-06

**Authors:** 


### Transformation of the Hydrate of Chloral into Chloroform in the Human Organism.

M. Personne's communication on Chloral, has just been brought before the Academy of Sciences. The results of his experiments point to the transformation of chloral into chloroform within the human organism. His experiments were conducted in two ways. First, chloral was directly mixed with the blood of an ox; next, the substance was given in toxic doses to a dog, which was slain when in a condition of complete anæsthesia. The blood of both animals was examined, and not the slightest trace of chloroform was to be detected by the smell. But when M. Personne, thinking that the smell of chloroform was necessarily covered by that of the blood, resorted to other means of investigation, he arrived at quite different results. A chemical analysis of the fluid was made, and then it was discovered, by employing the usual means for the detection of chloroform, that a large quantity of chloride of silver could be obtained from the blood which had been directly mixed up with the chloral, and a less quantity from the blood of the dog which had absorbed the substance. As the objection might be made that the chloride of silver does not result from the transformation of chloral and the consequent introduction of chloroform into the blood, M. Personne at once tells us that he could obtain the chloride of silver only when a small quantity of carbonate of sodium was added to the hydrate of chloral. Thus, he says, the alkali in this experiment has alone transformed chloral into chloroform, as does the alkali of the blood. The same results were obtained on examining the residue found in the stomach of the dog; and M. Personne states that absorption through this channel is extremely slow. Examination of the urine furnished no traces of chloroform—a fact which is in contradiction to what Dr. Bouchet has recently observed.

M. Personne has come to the conclusion "that hydrate of chloral does not pass through the human organism without undergoing a transformation, but that on reaching the blood it is separated into formic acid and cloroform, which latter substance is subsequently converted into chloride of sodium and formiate of soda, the products of its elimination."—*Lancet.*

